# *Trichosporon asahii* opportunistic pneumonia after a severe *COVID-19* infection^☆^^☆☆^

**DOI:** 10.1016/j.idcr.2023.e01701

**Published:** 2023-01-20

**Authors:** David S.J. Chang, Chawki W. Harfouch, Martha L. Melendez

**Affiliations:** Loma Linda University Murrieta Campus, Murrieta, CA, USA

**Keywords:** #COVID19 #trichosporonasahii #opportunisticinfection #fungal #cavitarypneumonia

## Abstract

This is the first reported case of fatal opportunistic *Trichosporon asahii* pneumonia in the setting of severe *COVID-19* pneumonia. The patient had ventilator-requiring respiratory failure secondary to *COVID-19* infection. The patient received intravenous broad-spectrum antibiotics, tocilizumab, and corticosteroids with subsequent development of cavitary infiltrates. Bronchoalveolar lavage grew *T. asahii*. We describe a rare complication of *COVID-19* infection and describe the microbial diagnosis, possible mechanism of infection, and optimal treatment.

## Introduction

Since 2019, *COVID-19* has affected a total of 96 million people with > 1 million deaths in the USA [1]. *COVID-19* patients are often placed on high dose corticosteroids and other immunosuppressants increasing the risk of opportunistic infections. Multiple fungal infections have been reported including pulmonary aspergillosis, mucormycosis, and invasive candidiasis [2]. We report the case of a 36-year-old patient with severe pneumonia complicated by fatal *T. asahii* pneumonia.

## Case

**Fig. 1:**
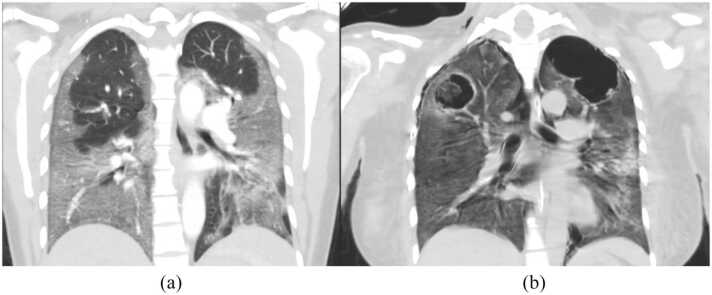
a. CT Chest with contrast on admission. b. CT chest without contrast on hospital day 20.

A 36-year-old *COVID-19* unvaccinated woman presented in April 2021, to a Southern California hospital for a five-day history of shortness of breath, cough, fever, and chills. She was diagnosed with *COVID-19* infection and was admitted for acute hypoxic respiratory failure requiring supplemental oxygen. Her past medical history was significant for a BMI of 28 and diet-controlled diabetes (hemoglobin A1c 6.3 %). She lived with her husband and two children and had no recent travel history. She reported vaping tobacco for 2 years and denied alcohol or recreational drug use. On admission, a chest CT angiogram showed bibasilar consolidations (Fig. 1-a). Laboratory results are shown in the Table 1.

**Table 1:** Comparison between hospital and ICU admission labs.

	Hospital admission lab	ICU admission lab	Reference ranges
WBC	14.78	51.39	4.8–11.8 bil/L
Hgb	12.9	10.7	10.5–15 g/dL
Plt	489	83	130–460 bil/L
Creatinine	0.4	3.8	0.7–1.3 mg/dL
AST	72	281	0–30 U/L
ALT	141	2390	7–37 U/L
Alk Phos	204	255	37–132 U/L
Total bilirubin	0.2	1.3	0–1.0 mg/dL
Lactate	2.3	16.8	0.5–2.0 mmol/L
C-reactive protein	3.9	0.8	0.7–1.3 mg/dL
Ferritin	1028	5773	12–300 ng/mL
Interleukin-6	80.9	1501	≤ 7.0 pg/mL

On hospital day 3, her oxygen requirement escalated to 60 liters by high-flow nasal cannula. She received a 5-day course of intravenous (IV) remdesivir, a 10-day course of dexamethasone and one dose of tocilizumab 400 mg. The patient's condition deteriorated and on hospital day eight she was transferred to the intensive care unit (ICU) and required intubation and mechanical ventilation. She developed multi-organ dysfunction syndrome with severe acute respiratory distress syndrome, septic shock, ischemic hepatitis, acute renal failure requiring hemodialysis, and left upper lobe pulmonary embolism. The patient received an empiric course of IV cefepime, metronidazole, vancomycin, and micafungin. Multiple blood cultures, bacterial and fungal sputum cultures, and urine cultures were negative. Serum aspergillus antigen and an interferon gamma assay were negative; beta-d-glucan was not available to order in the institution.

**Fig. 2:**
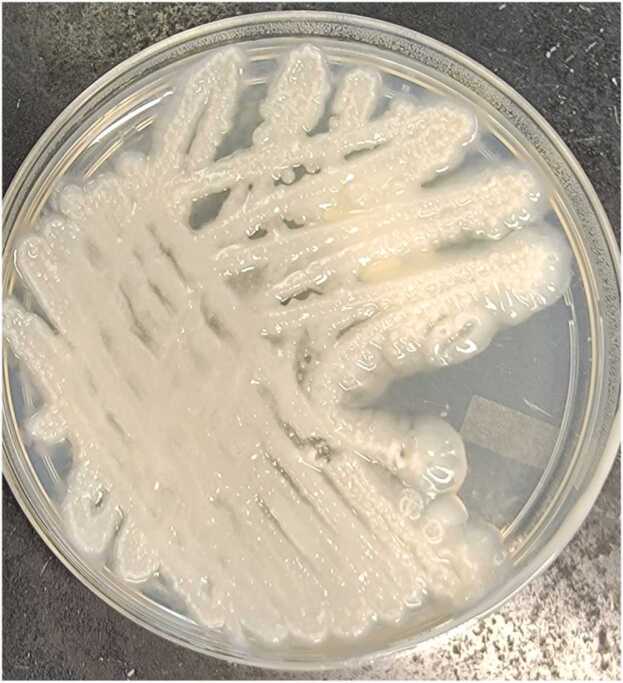
BAL fungal culture with *T. asahii* growing on Sabouraud dextrose agar. Courtesy of Mycology Laboratory, University of California, Irvine.

**Fig. 3:**
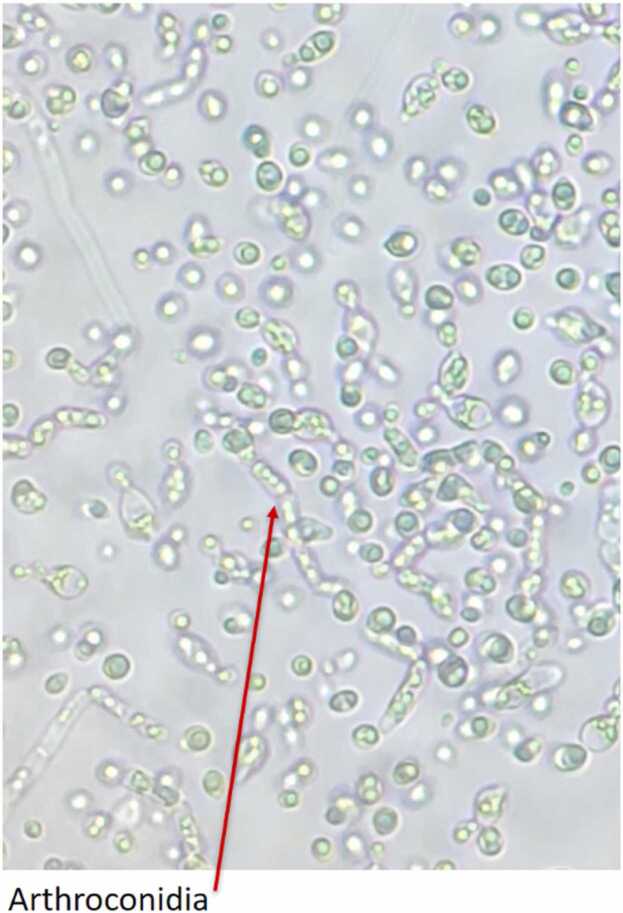
Wet mount demonstrating *T. asahii* arthroconidia. Courtesy of Mycology Laboratory, University of California, Irvine.

Despite broad antimicrobial therapy, the patient had a persistent leukemoid reaction (Table 1) and high oxygenation requirements. Repeat CT scan of the chest without contrast demonstrated bilateral upper lobe cavities which were not present on her prior CT scan (Fig. 1-b). Bacterial culture of endotracheal aspirate on day 20 was negative. Fungal culture grew yeast with hyphae and arthroconidia identified by MALDI- TOF as *T. asahii* (Fig. 2, Fig. 3).

On day 22, bronchoalveolar lavage (BAL) was performed yielding negative bacterial and mycobacterial cultures. BAL pneumocystis stain was negative. BAL fungal culture grew *T. asahii*. The patient was initially treated with IV liposomal amphotericin followed by voriconazole based on identification and susceptibility results. MICs were as follows: amphotericin (AMB) B 0.5 ug/mL, caspofungin > 8 ug/mL, fluconazole = 1 ug/mL, posaconazole = 0.125 ug/mL, voriconazole = 0.06 ug/mL, and itraconazole = 0.5 ug/mL. Despite aggressive medical therapy, the patient continued to deteriorate and expired on hospital day 37.

## Discussion

**Fig. 4:**
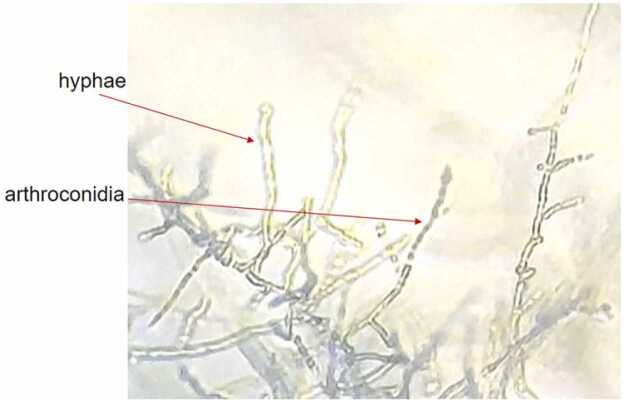
Microscopy of *T. asahii* grown on corn meal agar. Courtesy of Mycology Laboratory, University of California, Irvine.

*Trichosporon* is a urease-positive, non-encapsulated basidiomycetous environmental yeast characterized by the development of hyaline, septate hyphae that fragment into oval or rectangular arthroconidia. It is easily mistaken for candida but can be distinguished by the presence of arthroconidia (Fig. 4). *Trichosporon* is found in soil, water, and plants [3]. It could be part of the normal skin and gut flora. Human pathology ranges from benign superficial infection like white piedra to severe infections. Invasive Trichosporon infection is associated with immune deficiency (myeloproliferative neoplasms, organ transplants, AIDS, burn victims, catheter-acquired fungemia, and peritoneal dialysis catheter infections). It can disseminate into multiple organs and is associated with high mortality [4]. Since the 2019 novel coronavirus outbreak, there have been several reports of *T. asahii* infection in severe *COVID-19* cases. A study in Brazil described invasive *Trichosporon* opportunistic infections in the setting of *COVID-19*. The study conducted between July and September 2020 in a single hospital described five *T. asahii* fungemia cases. Risk factors were older age, morbid obesity, prolonged corticosteroid use, previous broad-spectrum antibiotics and echinocandin exposure [5]. Another study describes nine cases of *Trichosporon* and *COVID-19* coinfection in the world: 6 in Brazil, one in the United States, one in Qatar, and one in Spain. All nine patients had severe *COVID-19*, received broad-spectrum antibiotics and systemic corticosteroids [6].

Severe alveolar damage caused by ARDS could be a predisposing risk factor for invasive pulmonary disease. An autopsy performed on a triple pulmonary coinfection with *T. asahii*, *Pseudomonas aeruginosa*, *and Stenotrophomonas maltophila* showed diffuse alveolar damage with severe inflammatory exudates [7]. A Hong Kong study intranasally challenged 8 golden hamsters (*Mesocricetus auratus*) with the *COVID-19* virus following them for 14 days and obtaining serial viral loads and histopathologic studies postmortem. Most hamsters had a large number of *COVID-19* nucleocapsid proteins in enteric epithelial cells and developed severe epithelial cell necrosis, damaged intestinal villi, and mucosal inflammation [8]. We hypothesize that the pathogenesis of invasive fungal infections in the setting of severe *COVID-19* could be the result of mucosal injury and immune barrier disruption combined with the dysbiosis caused by broad-spectrum antibiotics. Furthermore, *COVID-19* patients are typically immunosuppressed secondary to corticosteroid and immunomodulator use causing susceptibility to opportunistic infections [5], [6].

The 2014 guidelines from European Society of Clinical Microbiology and Infectious Diseases (ESCMID) and European Confederation of Medical Mycology (ECMM) recommend treatment with voriconazole based on in vitro studies as *T. asahii* is often resistant to amphotericin B (MIC > 2 mg/L) and caspofungin (MIC > 16 mg/L) [9]. The MIC breakpoints for *T. asahii* species are not yet established. Therefore, the breakpoints were extrapolated from Candida albicans as per the CLSI guidelines. A multicenter retrospective Taiwanese study aggregated MIC data from 84 patients between 2010 and 2018 showed MIC range as following: Voriconazole (0.06–0.25 μg/mL), Micafungin (> 8 μg/mL), Amphotericin B 0.12–2 (μg/mL), and fluconazole (2–16 μg/mL). This data suggests that *T. asahii* is most susceptible to voriconazole [10].

## Conclusion

To our knowledge, this is the first report of fatal pneumonia due to *T. asahii* as the sole microbial pathogen in the setting of severe *COVID-19* infection. *T. asahii* has been linked to fungemia in critically ill *COVID-19* patients, but pneumonia is rare. The delayed identification of *T. asahii* and the common use of echinocandins may contribute to increased morbidity and mortality. More clinical data are needed to optimize the diagnosis and treatment of severe *Trichosporon* infections.

## CRediT authorship contribution statement

**Chang, David Sea Jong:** Conceptualization, Investigation, Writing – original draft, Writing – review & editing, Visualization. **Harfouch, Chawki W:** Conceptualization, Investigation, Writing – original draft, Writing – review & editing. **Melendez, Martha L.:** Writing – review & editing.

## Ethical approval

No intervention was done to the patient and no ethical dilemma identified.

## Consent

Pt is deceased and obtained consent from her late husband.

## Funding

All authors involved in this study did not receive any external or internal funding.

## Conflict of Interest

All authors involved in this study have no conflict of interest.
